# SARS-COV-2 Infection in Children and Red Blood Cell Distribution Width

**DOI:** 10.7759/cureus.17837

**Published:** 2021-09-09

**Authors:** Hyppolite Tchidjou Kuekou, Lucia Palandri, Suzanne Pouplin, Valerie LiThiao Te, Elena Righi, Sandrine Castelain, Jannick Ricard

**Affiliations:** 1 Pediatric Emergency Services, Amiens University Hospital, Amiens, FRA; 2 Department of Biomedical, Metabolic and Neural Sciences, University of Modena and Reggio Emilia, Modena, ITA; 3 Department of Pediatric Hematology/Oncology, Amiens University Hospital, Amiens, FRA; 4 Department of Microbiology, Amiens University Hospital, Amiens, FRA

**Keywords:** sars-cov-2 in children, red blood cell distribution width (rdw), erythrocyte indices, covid-19, severity of disease, prognostic role, pediatrics emergency, clinical outcomes, covid-19 outcome predictor

## Abstract

SARS-COV-2 infection due to Coronavirus is highly contagious and causes varying degrees of illness throughout the world. Recent literature has shown an association between red blood cell distribution width (RDW) and adverse outcomes among adult patients with COVID‐19. Multiple hypotheses can explain the potential prognostic role of RDW in COVID-19 infection.

The aim of this study is to describe RDW values in SARS-COV-2 infected children admitted to the Pediatric Emergency Department to shed light on the potential role of RDW as a prognostic factor in this specific group.

Of 1086 tested children observed from March 2020 to April 2021, 36 positive SARS-COV-2 children (0-16 years) did not show clinically significant differences in RDW values according to illness categories, days of hospitalization, presence of multisystem inflammatory syndrome in children (MIS-C), or viral load (cycle threshold (CT) values).

This study is the first to investigate this issue in a SARS-COV-2 infected pediatric population.

Despite our negative results, given the high incidence of Delta variant in children, the low cost of the examination, its prognostic role described in adults, and its association to other pediatric illnesses, we believe that the role of RDW in SARS-COV-2 infected children should be deeper assessed and that larger collaborative studies on this issue are required.

## Introduction

As recently discussed by Bommenahalli Gowda et al. [1], there seems to be an association between red blood cell distribution width (RDW) and adverse outcomes among patients with COVID‐19. Consistent with this, Henry et al. [2] suggested the utility of measuring RDW as a single point prognostic factor for adults admitted in the emergency departments (ED). Only patients older than 16 years old with SARS-COV-2 infections were included in the studies reviewed by Lee et al. [3].

RDW is a measure of the range of variation of red blood cell (RBC) volume that is reported as part of a standard complete blood count. The identification of early markers of disease severity would greatly facilitate the selection of COVID-19 patients requiring more aggressive monitoring and management, as well as appropriate use of healthcare resources [4]. Multiple hypotheses can explain the potential prognostic role of RDW in COVID-19. Conditions that have an impact on erythrocyte’s biology and consequently anisocytosis may be secondary to indirect RBC damage consequent to hemolytic anemia or intravascular coagulopathy, direct damage due to infection of erythrocytes or their bone marrow precursors, and an iron metabolism perturbation caused by sustained inflammatory response [2].

SARS-COV-2 positive children evaluated in ED rarely have notable disease symptoms, yet they represent an organizational burden. Nevertheless, some of them show rare and life-threatening presentations and questions remain open regarding the cause and the severity of clinical manifestation in the pediatric population. The true incidence of severe disease in children, such as multisystem inflammatory syndrome in children (MIS-C), remains unclear. A clinical and laboratory bedside approach could be more useful than a radiological one in predicting child management and allowing a better resource allocation [5].

In literature, there are little data regarding RDW and its potential clinical impact in children, yet elevated RDW values show to play a role as an early pragmatic biomarker for outcomes in pediatric critical illnesses as neonatal sepsis, critically ill children, EBV infection, serious bacterial infection, and inflammatory bowel disease [6-10].

The aim of this work is therefore to describe RDW values in a SARS-COV-2 infected pediatric population admitted in the Pediatric Emergency Departments (PED) to shed light on the potential role of RDW as a prognostic factor.

## Materials and methods

We performed a cross-sectional study including children who tested positive for SARS-COV-2 in the PED of a University Hospital, Amiens-France during the last year.

Patients aged 0 to 16 years old accessed by the PED with COVID-19 suggestive symptoms or identified as contact of a SARS-COV-2 positive case or who needed to be admitted for other causes, received a nasopharyngeal swab. If positive, data on demographics, clinical symptoms at presentation, clinical outcomes (hospitalization and mortality), viral load (cycle threshold (CT) values), and available RDW values were collected. Patients were divided into illness categories as shown in Table 1, according to the NIH classification [11]. Due to the small number of subjects, only descriptive statistical analysis of data was performed.

**Table 1:** RDW values in 36 SARS-CoV-2 infected children stratified by clinical characteristics and n(%) above and below RDW threshold °: % on overall (36), †: % of row. *CT: cycle threshold (low: CT<20, moderate: 21<CT<30, high>31).

	n (%)	Median	Range	RDW < 15%^†^	RDW ≥ 15%^†^
Overall	36 (100%)	12.8	11.4–16.4	32 (89%)	4 (11%)
Illness categories					
Asymptomatic	2 (6%)	12.5	12.5–12.5	2 (100%)	0 (0%)
Mild illness	24 (67%)	12.9	11.7–16.2	21 (88%)	3 (13%)
Moderate illness	3 (8%)	13.0	12.7–13.2	3 (100%)	0 (0%)
Severe illness	6 (17%)	13.4	11.9–16.4	5 (83%)	1 (17%)
Critical illness	1 (3%)	11.4	11.4–11.4	1 (100%)	0 (0%)
Hospitalized					
Yes	30 (83%)	12.9	11.4–16.4	27 (90%)	3 (10%)
No	6 (17%)	12.7	11.7–16.2	5 (83%)	1 (17%)
MIS-C					
Yes	1 (3%)	11.4	11.4–11.4	1 (100%)	0 (0%)
No	35 (97%)	12.8	11.7–16.4	31 (89%)	4 (11%)
Viral load CT*					
Low	9 (25%)	12.4	11.4–16.2	8 (89%)	1 (11%)
Moderate	8 (22%)	14.0	12.8–15.3	7 (88%)	1 (13%)
High	12 (33%)	12.6	11.8–16.4	11 (92%)	1 (8%)

The study is a focus of a major study that was approved by the local ethics committee with the protocol number: PI2021_843_0052 and performed in the respect of the Helsinki declaration. Routine clinical data were collected in an anonymized format.

Before completing the present study, a scoping review of the literature was performed to assess current evidence on the use of RDW as a prognostic factor in SARS-COV-2 infected children.

## Results

Of 1086 tested children observed from March 2020 to April 2021, 48 (4.4%) tested positive for SARS-COV-2. Twenty-two (46%) were female, the median age was 19.5 months (25°:4, 75°:119, range: 0-202 months). At presentation, 10% were asymptomatic, 67% had a mild illness, 8% moderate illness, 13% severe illness, and one presented with MIS-C in critical conditions; 32 (67%) children were hospitalized for a median length of three days (25°:1.5, 75°:5.5, range: 1-25 days); all patients survived. RDW values at presentation were available for 36 patients (75%). Table 1 describes RDW values throughout our sample and complete descriptive statistical analysis is given in Table 2.

**Table 2:** Full statistical descriptive analysis of RDW values in 36 SARS-COV-2 infected children stratified by clinical characteristics and n(%) above and below RDW threshold : % on overall (36), †: % of row. *CT: cycle threshold (low: CT<20, moderate: 21<CT<30, high>31).

	n (%)	Mean	SD	Median	25°	75°	Range	IQR	RDW < 15%^†^	RDW ≥ 15%^†^
Overall	36 (100%)	13.1	1.3	12.8	12.3	13.8	11.4–16.4	1.6	32 (89%)	4 (11%)
Illness categories										
Asymptomatic	2 (6%)	12.5	NA	12.5	NA	NA	12.5–12.5	NA	2 (100%)	0 (0%)
Mild illness	24 (67%)	13.2	1.3	12.9	12.3	13.8	11.7–16.2	1.55	21 (88%)	3 (13%)
Moderate illness	3 (8%)	13.0	0.3	13.0	12.7	13.2	12.7–13.2	0.5	3 (100%)	0 (0%)
Severe illness	6 (17%)	13.6	1.7	13.4	12.1	14.8	11.9–16.4	2.7	5 (83%)	1 (17%)
Critical illness	1 (3%)	11.4	NA	11.4	NA	NA	11.4–11.4	NA	1 (100%)	0 (0%)
Hospitalized										
Yes	30 (83%)	13.1	1.2	12.9	12.4	13.6	11.4–16.4	1.3	27 (90%)	3 (10%)
No	6 (17%)	13.2	1.7	12.7	11.8	14.8	11.7–16.2	3	5 (83%)	1 (17%)
MIS-C										
Yes	1 (3%)	11.4	NA	11.4	NA	NA	11.4–11.4	NA	1 (100%)	0 (0%)
No	35 (97%)	13.2	1.3	12.8	12.4	13.9	11.7–16.4	1.5	31 (89%)	4 (11%)
Viral load CT*										
Low	9 (25%)	12.7	1.5	12.4	11.8	13.3	11.4–16.2	1.5	8 (89%)	1 (11%)
Moderate	8 (22%)	13.8	0.9	14.0	12.9	14.3	12.8–15.3	1.4	7 (88%)	1 (13%)
High	12 (33%)	12.9	1.3	12.6	12.2	13.0	11.8–16.4	0.8	11 (92%)	1 (8%)

Above normal range values (RDW>15%) were observed for four patients: they were all male, all European with a median age of 1.5 months (25°:0, 75°:7, range: 0-22 months). Among them, three presented with mild symptoms, one with severe, and three were hospitalized in the ordinary ward for surveillance, for a median of five days. Of seven patients who presented with severe or critical illness, only one showed above normal range values for RDW. Data show an inverse association between RDW and age as described in the literature [12] and no other clinically significant association.

**Figure 1:**
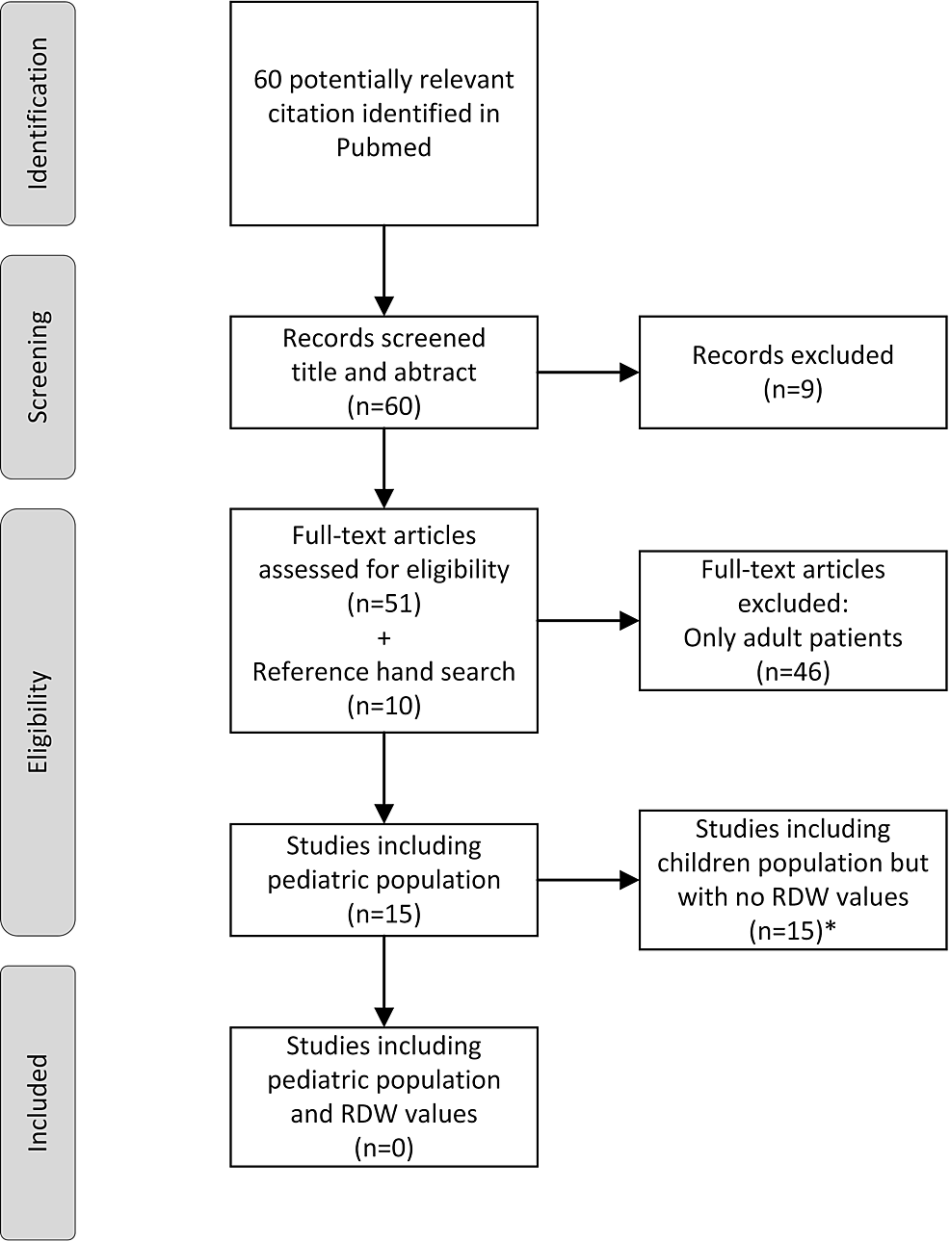
Scoping review on RDW in SARS-COV-2 infected children, a flowchart of included studies *Excluded articles had no RDW values or presented values only in aggregated form with the entire adult cohort

A systematic literature search was performed in PubMed, supplemented by a hand search of references from relevant publications. The search for identifying qualifying studies was initiated by constructing sets of relevant keywords (i.e., RDW and COVID‐19) and their synonyms. These search terms were expanded and organized in thematic building blocks, as provided in Appendices. Eligible studies were identified as the presence of RDW values referring to a SARS-COV-2 infected pediatric population (0-18 years old). Nonoriginal publications (e.g., narrative review, systematic review, meta‐analysis, editorial) were included and a reference search was performed for potentially eligible studies. Of 60 screened articles, none reported RDW values in SARS-COV-2 infected pediatric population (Figure 1).

## Discussion

The present study is a cross-sectional clinical investigation of the diagnostic value of increased RDW in children with SARS-COV-2 infection. Sampling RDW at admission in the emergency department has been shown to have a strong prognostic power in adults [2], however, to the best of our knowledge, this is the first study in the literature that investigates this issue in SARS-COV-2 infected children admitted for different reasons in a Pediatric Emergency Department.

Given that, the majority of SARS-COV-2 infected children are asymptomatic or present with mild symptoms, blood samples are not often taken thus data blood tests, as RDW, are limited.

In healthy newborns, RDW has been shown to be markedly elevated, ranging from 14.2% to 17.8% during the first 30 days of life, because of elevated anisocytosis and poikilocytosis. The number of “dysmorphic” red blood cells is even higher in premature infants. After that, it gradually decreases and reaches normal adult levels by six months of age [12,13].

Then, the hemopoietic system, especially the marrow cellularity, is progressively affected by aging, however, its effects become notable after age 65 years. Marrow cellularity begins at 80-100% in infancy and declines up to 30% after age 65 years. This suggests why RDW could be a stronger prognostic factor in COVID-19 infected adults (given that severe outcomes are more frequent in elders) than in infants, in whom the bone marrow is able to rapidly replace defective RBC. This has been said, the membrane of newborn’s red cells is different from adults making them more susceptible to osmotic hemolysis and oxidant injury than are adult cells [12,13]. In SARS-COV-2 infected children, the low prevalence of sustained inflammatory and hematological critical responses results in the fact that RDW has not been up to now a considered parameter as a prognostic factor for severe outcomes.

In contrast to what observed in adults [1-3], our results do not suggest the existence of clinically significant differences in RDW values in children (0-16 years) according to illness categories, hospitalization, and presence of MIS-C, days of hospitalization or viral load (CT values).

However, our negative results could be related to some pitfalls of our study. First, the low prevalence of severe outcomes in the pediatric population and the limited sample size of our cohort may underestimate the power of RDW as a prognostic factor. Second, as the majority of SARS-COV-2 infected children are asymptomatic or present with mild symptoms, blood samples are not always taken and thus data on blood tests, such as RDW, were not available for all positive children. Moreover, an important limitation to our study is given by the fact that patients with chronic disease may have direct access to the clinical ward responsible for their follow-up without passing through the PED.

Given the new epidemic wave due to the SARS-COV-2 Delta variant, with a higher incidence in children than previous variants, it is important to identify an early and easily accessible laboratory prognostic factor, such as RDW which could facilitate the selection of COVID-19 children requiring close monitoring and follow-up. Yet, as in our study only 4 patients out of 1086 screened had a positive result, we deem it important to encourage colleagues who wish to investigate this issue, to further evaluate it with larger collaborative studies.

## Conclusions

In conclusion, in this work, a prognostic role of RDW in pediatric SARS-COV-2 infection was not observed, as this parameter result was not associated with any of the measured variables. Despite this, given the high incidence of Delta variant in children, the low cost of the examination, its prognostic role described in adults with COVID-19, and its association to other pediatric illnesses, we believe that the role of RDW in SARS-COV-2 infected children should be deeper assessed and further studies on this issue are required.

## Appendices

**Table 3:** Search strategy for scoping review

Query in PubMed
("covid 19"[All Fields] OR "covid 19"[MeSH Terms] OR "covid 19 nucleic acid testing"[All Fields] OR "covid 19 nucleic acid testing"[MeSH Terms] OR "covid 19 serological testing"[All Fields] OR "covid 19 serological testing"[MeSH Terms] OR "covid 19 testing"[All Fields] OR "covid 19 testing"[MeSH Terms] OR "sars cov 2"[All Fields] OR "sars cov 2"[MeSH Terms] OR "severe acute respiratory syndrome coronavirus 2"[All Fields] OR "ncov"[All Fields] OR "2019 ncov"[All Fields] OR (("coronavirus"[MeSH Terms] OR "coronavirus"[All Fields] OR "cov"[All Fields]) AND 2019/11/01:3000/12/31[Date - Publication])) AND ("erythrocyte indices"[MeSH Terms] OR ("erythrocyte"[All Fields] AND "indices"[All Fields]) OR "erythrocyte indices"[All Fields] OR ("red"[All Fields] AND "cell"[All Fields] AND "distribution"[All Fields] AND "width"[All Fields]) OR "red cell distribution width"[All Fields])
Searched: 1 July 2021
